# The impact of exercise on cognitive function and brain health across the lifespan: A systematic review

**DOI:** 10.1002/ibra.70024

**Published:** 2026-06-14

**Authors:** Issam AbuQeis, Abeer Teeti, Amro Titi

**Affiliations:** ^1^ Department of Radiology Palestinian Ministry of Health Ramallah Palestine; ^2^ Institute of Neuroscience Kunming Medical University Kunming China; ^3^ Department of Chemistry Hebron University Hebron Palestine; ^4^ Department of Epidemiology, School of Public Health Kunming Medical University Kunming China; ^5^ Department of Internal Medicine Palestinian Ministry of Health Ramallah Palestine

**Keywords:** brain health, cognitive function, exercise, neuroplasticity, PRISMA

## Abstract

Exercise has profound positive effects on cognitive function and brain health throughout different life stages. This systematic review aims to evaluate the multifaceted impacts of exercise on cognitive function, neuroplasticity, neurotransmitter expression, cerebrovascular function, and age‐related cognitive decline across the lifespan. Following Preferred Reporting Items for Systematic Reviews and Meta‐Analyses (PRISMA) 2020 guidelines, we systematically searched PubMed, Web of Science, and Scopus databases for studies examining exercise interventions and cognitive function. Eligibility criteria included studies involving human participants across all age groups and animal models focusing on molecular mechanisms. The review employed qualitative synthesis to examine exercise modalities and age‐specific effects. Risk of bias was assessed using the Joanna Briggs Institute Critical Appraisal Checklists. A total of 37 studies were included in the final synthesis (young adults: 2, middle‐aged adults: 7, older adults/neurodegenerative diseases: 13, molecular mechanisms: 15). Regular physical activity, including aerobic, resistance, and combined training, significantly enhances cognitive abilities, promotes neuroplasticity, and mitigates age‐related cognitive decline. Key molecular mechanisms include upregulation of brain‐derived neurotrophic factor (BDNF) and vascular endothelial growth factor (VEGF), and modulation of dopaminergic and serotonergic systems. Exercise serves as a potent non‐pharmacological intervention for maintaining cognitive vitality and mental health. Limitations include heterogeneity in exercise protocols and outcome measures across studies. Future research should focus on multi‐omics approaches to further elucidate cell‐type‐specific effects.

## Graphical abstract

This systematic review of 37 studies demonstrates that aerobic, resistance, and combined exercise enhance cognitive function and brain health across all ages. Exercise promotes neuroplasticity, upregulates brain‐derived neurotrophic factor (BDNF) and vascular endothelial growth factor (VEGF), and mitigates age‐related cognitive decline, establishing physical activity as a powerful non‐pharmacological intervention for lifelong neurological well‐being.

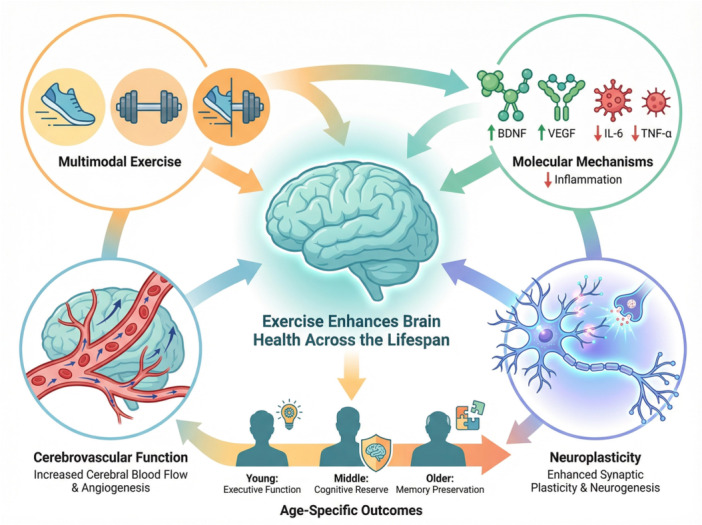

## 1 INTRODUCTION

Exercise has emerged as a powerful non‐pharmacological intervention for enhancing cognitive function and promoting brain health across the lifespan. Extensive research has demonstrated that regular physical activity positively impacts neuroplasticity, neurogenesis, and overall brain structure and function.^1^ For instance, studies have shown that aerobic exercise increases hippocampal volume and neurogenesis in both young and older adults.^1^, ^2^ Exercise also upregulates the expression of neurotrophic factors such as brain‐derived neurotrophic factor (BDNF), which supports neuron survival and synaptic plasticity.^2^ Furthermore, exercise has been found to improve cerebrovascular function by promoting angiogenesis and enhancing cerebral blood flow, which is crucial for maintaining cognitive health.^3^ In young adults, exercise enhances cognitive flexibility, attention, and executive function, even with short bouts of activity.^4^ These cognitive benefits are supported by neurophysiological changes such as increased BDNF levels and improved prefrontal cortex activation during complex tasks.^2^ Regular physical activity in this population not only improves academic and work performance but also establishes healthy habits that can mitigate cognitive decline later in life.

Middle‐aged adults may experience the neuroprotective effects of exercise by building cognitive reserve, which helps delay the onset of age‐related cognitive impairment. Research indicates that higher cardiorespiratory fitness (CRF) in middle age is associated with greater gray matter volume in brain regions vulnerable to aging, such as the middle temporal gyrus and perirhinal cortex.^3^ Exercise during this life stage may also attenuate the progression of white matter hyperintensities, which are markers of small vessel disease and cognitive decline.^5^ For older adults, exercise is a critical factor in maintaining cognitive function and reducing the risk of neurodegenerative diseases. Long‐term aerobic exercise has been shown to improve memory and spatial cognition in older adults by enhancing synaptic plasticity and reducing neuroinflammation.^6^ Resistance training also plays a role in cognitive health by increasing myokines such as interleukin‐6 (IL‐6) and insulin‐like growth factor 1 (IGF‐1), which support neuroplasticity and muscle‐brain interactions.^7^ Combined exercise programs that include both aerobic and resistance training have demonstrated synergistic effects on cognitive and physical health, highlighting the importance of multimodal approaches in this population.

The neuroprotective mechanisms of exercise are multifaceted. At the molecular level, exercise modulates inflammatory pathways by downregulating pro‐inflammatory cytokines such as tumor necrosis factor alpha (TNF‐α) and interleukin‐1β while upregulating anti‐inflammatory cytokines like interleukin‐10 (IL‐10).^8^ Exercise also activates signaling pathways involved in neuronal survival and plasticity, such as the BDNF‐TrkB pathway and the PI3K/AKT pathway.^9^ Additionally, exercise‐induced improvements in cerebrovascular function, including increased cerebral blood flow and angiogenesis, contribute to the maintenance of cognitive health.^3^ This systematic review aims to provide a comprehensive overview of the current evidence regarding the effects of exercise on cognitive function and brain health across different age groups and various types of exercise. We will explore the underlying mechanisms through which exercise exerts its beneficial effects and discuss future research directions to optimize exercise interventions for cognitive enhancement and neuroprotection. Previous systematic reviews and meta‐analyses have examined the relationship between exercise and cognition within specific populations. For example, Northey et al., conducted a meta‐analysis demonstrating that physical exercise improves cognitive function in adults over 50,^10^ while Lambourne and Tomporowski synthesized evidence on the acute effects of exercise on cognition.^11^ However, comprehensive reviews that integrate evidence across the entire lifespan, from young adults to older adults with neurodegenerative diseases, while simultaneously examining the underlying molecular mechanisms, remain scarce. The present review addresses this gap by synthesizing findings across all age groups and incorporating recent advances in omics‐based approaches to elucidate cell‐type‐specific neuroprotective pathways.

## 2 METHODS

This systematic review was conducted and reported in accordance with the Preferred Reporting Items for Systematic Reviews and Meta‐Analyses (PRISMA) 2020 guidelines.^12^ This systematic review was not prospectively registered in a protocol registry (e.g., PROSPERO). However, the review methodology was developed a priori and strictly adhered to the predefined eligibility criteria, search strategy, and synthesis methods described below.

### 2.1 Eligibility criteria

Studies were selected based on the following population, intervention, comparison, and outcome (PICO) criteria. Population: Individuals across the lifespan (young adults, middle‐aged adults, older adults) and animal models utilized to study relevant molecular mechanisms of aging and neurodegeneration. Intervention: Any form of physical exercise intervention, including aerobic exercise, resistance training, high‐intensity interval training (HIIT), and combined multimodal exercise programs. Comparison: Control groups including no intervention, sedentary lifestyle, or alternative non‐physical interventions. Outcomes: Primary outcomes included cognitive function (memory, executive function, attention, cognitive flexibility), brain structural changes (gray matter volume, white matter integrity), and molecular biomarkers (BDNF, vascular endothelial growth factor [VEGF], inflammatory cytokines, neurotransmitters). As for study design, randomized controlled trials (RCTs), quasi‐experimental studies, and longitudinal cohort studies were included. Reviews, editorials, and non‐peer‐reviewed articles were excluded.

### 2.2 Information sources

A comprehensive literature search was conducted across three major databases: PubMed, Web of Science, and Scopus. The search encompassed literature from Jan 2010 to Dec 2025. Reference lists of included studies and relevant review articles were also manually screened to identify additional eligible studies. We did not search gray literature databases, trial registries (e.g., ClinicalTrials.gov, WHO ICTRP), or dissertation repositories; consequently, unpublished negative or null findings may not be fully represented.

### 2.3 Search strategy

The search strategy was developed using a combination of Medical Subject Headings (MeSH) and free‐text keywords related to “exercise,” “cognitive function,” and “lifespan.” The full search string for PubMed is provided in the supplementary materials (Supplementary S1) and includes terms such as (“Exercise”[MeSH] OR “Physical Activity”) AND (“Cognition”[MeSH] OR “Cognitive Function” OR “Brain Health”) AND (“Age Groups”[MeSH] OR “Lifespan”). Appropriate syntax adaptations were made for Web of Science and Scopus.

### 2.4 Selection process

Two independent reviewers screened the titles and abstracts of all retrieved records. Full‐text articles of potentially eligible studies were then retrieved and independently assessed for final inclusion based on the predefined eligibility criteria. Any discrepancies between the two reviewers were resolved through discussion and consensus, or by consulting a third independent reviewer when necessary.

### 2.5 Data collection process and data items

Data extraction was performed independently by two reviewers using a standardized, pre‐piloted data extraction form. Extracted data items included: study characteristics (author, year, study design), participant demographics (age group, sample size, health status), intervention details (type, intensity, duration, frequency), comparator, and key findings related to cognitive and neurobiological outcomes.

### 2.6 Study risk of bias assessment

The methodological quality and risk of bias of the included studies were assessed using the appropriate Joanna Briggs Institute (JBI) Critical Appraisal Checklists (Supplementary S2) based on the study design. Two reviewers independently conducted the quality appraisal, with disagreements resolved by a third reviewer. The results of the risk of bias assessment were used to inform the synthesis and interpretation of the findings.

### 2.7 Synthesis methods

Due to substantial clinical and methodological heterogeneity across the included studies, including wide variation in exercise protocols (acute single bouts to 6‐month interventions), diverse cognitive assessment instruments (e.g., Mini‐Mental State Examination [MMSE], Montreal Cognitive Assessment [MoCA], computerized batteries, animal behavioral tasks), and mixed study populations (healthy adults, clinical populations, animal models), a formal meta‐analysis was not deemed appropriate. Partial meta‐analyses within more homogeneous subgroups were considered; however, the variability in outcome measures and intervention parameters within each subgroup remained too great to permit meaningful pooling. Instead, a narrative synthesis of the findings was conducted. The magnitude and direction of treatment effects were assessed qualitatively based on the statistical significance, effect sizes (where reported), and consistency of findings across studies within each thematic category. The results were thematically organized into sections based on age groups (young adults, middle‐aged adults, older adults), specific neurodegenerative diseases (Alzheimer's Disease [AD], Parkinson's Disease [PD]), and underlying molecular mechanisms. The PRISMA 2020 checklist was shown in Supplementary S3.

## 3 RESULTS

### 3.1 Study selection

**Figure 1:**
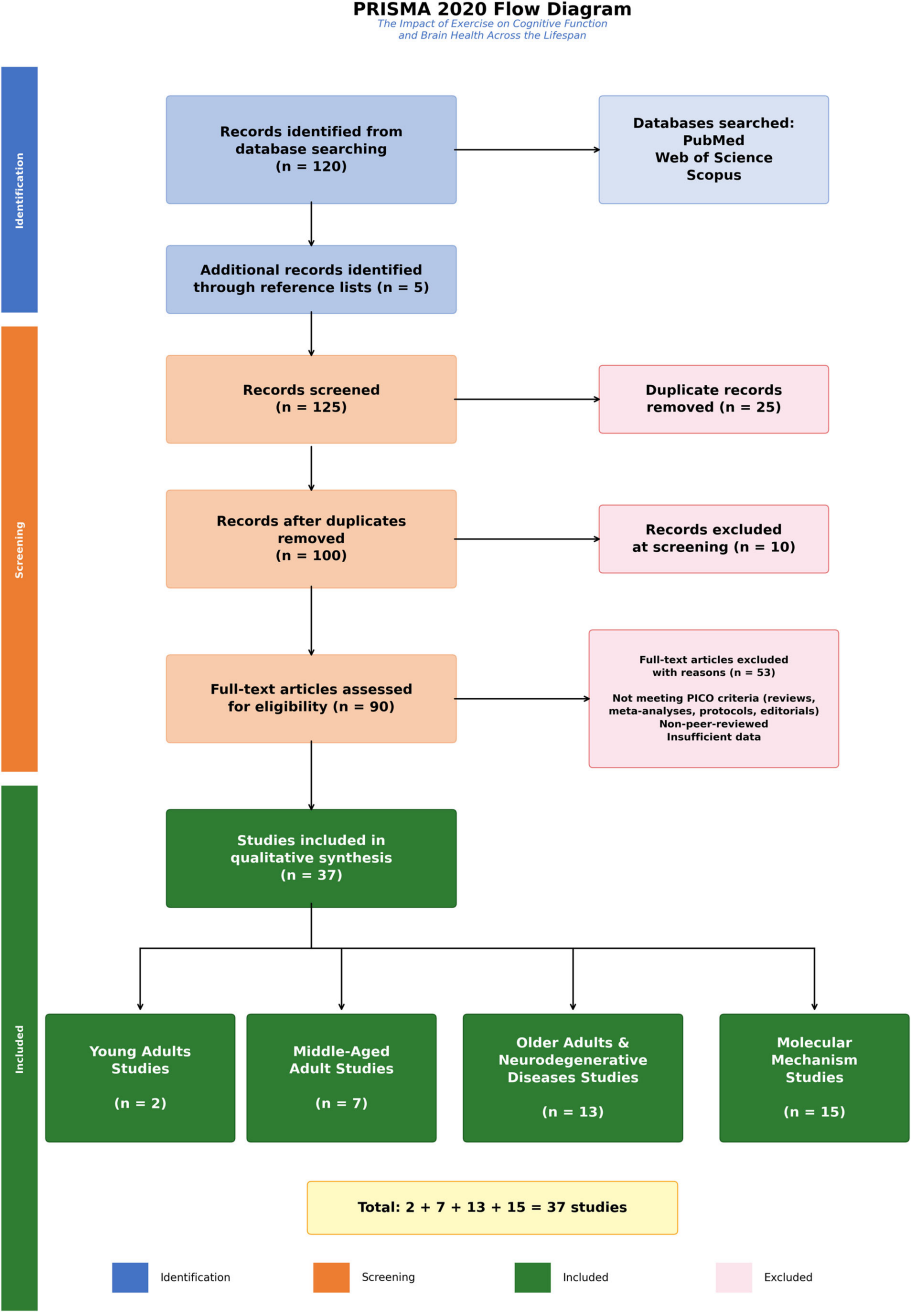
PRISMA 2020 flow diagram of study selection process.

The initial database search yielded 120 records. After removing duplicates and screening titles and abstracts, 37 studies met the inclusion criteria: young adults (2 studies), middle‐aged adults (7 studies), older adults and neurodegenerative diseases (13 studies), and molecular mechanism studies (15 studies). Of the 37 included studies, 21 utilized animal models, while the remaining 16 were conducted in human participants or utilized human biological samples (see Supplementary S4 for study characteristics). A PRISMA flow diagram detailing the selection process is provided in Figure 1.

### 3.2 Quality appraisal

The overall methodological quality of the included studies was moderate to high, as assessed by the JBI Critical Appraisal Checklists. Most RCTs clearly described randomization procedures and outcome measurements. Quasi‐experimental and animal studies generally demonstrated appropriate control groups and reliable outcome assessments. Detailed quality evaluation and evidence synthesis are provided in the supplementary materials (Supplementary S2 and S4).

### 3.3 Types and intensity of exercise

#### 3.3.1 Aerobic exercise

Aerobic exercise, such as treadmill running, improves cognitive function in both the short and long term. Sixteen weeks of treadmill exercise improved spatial learning and memory in aged rats by increasing hippocampal neurogenesis and synaptic plasticity with increased white matter volume and the total length of microvessels.^13^ Short‐term aerobic exercise increases BDNF levels, crucial for cognitive function.^2^ Moderate‐intensity aerobic exercise enhances memory and reduces neuroinflammation,^6^ while HIIT improves cognitive flexibility and reduces anxiety.^14^ 12 weeks of aerobic exercise improved brain energy metabolism in mild AD patients by increasing ketone uptake.^15^

#### 3.3.2 Resistance training

Resistance training improves muscle function and enhances cognitive function. It increases BDNF levels and improves memory,^16^ and upregulates myokines such as IL‐6, IGF‐1, and BDNF, enhancing cognitive function and skeletal muscle health in older adults.^7^ Resistance training regulates neuromuscular pathways, upregulating neurotrophic factors and reducing oxidative stress.^16^

#### 3.3.3 Combined exercise programs

Combined exercise programs (aerobic, resistance, and cognitive training) have synergistic effects. A combination of treadmill exercise and cognitive training improved gait and cognitive function in PD patients.^17^ Meta‐analytic evidence further confirms that dual‐task training improves gait speed, balance, and motor function in PD compared with single‐task training or usual care. In early‐stage PD, combined aerobic and resistance training has also been shown to enhance cognitive processing speed beyond aerobic exercise alone,^18^ and head‐to‐head comparisons in human RCTs have suggested that combined aerobic and resistance training may yield greater cognitive benefits than either modality alone,^19^ supporting the synergistic hypothesis.

### 3.4 Effects of exercise on cognitive functions across age groups

#### 3.4.1 Young adults

Aerobic exercise exerts significant positive effects on cognitive function in young adults, particularly in the domains of cognitive flexibility, attention, and executive function. Exercise‐induced increases in BDNF contribute to enhanced synaptic plasticity,^2^ thereby supporting cognitive adaptability and learning processes. Studies have shown that young adults who demonstrated improved cognitive task performance during dual‐task walking also exhibited reduced frontal electroencephalographic (EEG) activity, suggesting a more efficient reallocation of cognitive resources and enhanced inhibitory control.^20^ In addition, even a brief 10‐min session of aerobic exercise has been reported to improve executive function in both young and older adults, regardless of exercise intensity or baseline physical fitness levels.^4^, ^21^ These improvements in attention and executive functioning may further translate into enhanced academic and occupational performance by facilitating problem‐solving abilities, sustained focus, and multitasking capacity.^21^ Furthermore, the cognitive benefits associated with dual‐task walking indicate improved cognitive flexibility, which may support more effective management of simultaneous tasks in daily life.^20^

#### 3.4.2 Middle‐aged adults

In middle‐aged adults, aerobic exercise has been shown to significantly enhance cognitive reserve and cardiovascular health. Higher CRF is associated with greater volume in the middle temporal gyrus and perirhinal cortex,^3^ as well as increased gray matter volume in the frontal cortex, which is particularly beneficial for individuals with amnestic mild cognitive impairment.^22^ Furthermore, a 3‐month aerobic exercise program has been demonstrated to improve brain energy metabolism.^15^ Regarding cardiovascular health, higher CRF attenuates age‐related increases in white matter hyperintensities (WMH).^5^ Notably, 6 weeks of treadmill exercise improved cognitive function in middle‐aged mice with subcortical ischemic vascular dementia.^23^

#### 3.4.3 Older adults

Exercise interventions exert profound effects on memory, spatial cognition, and neuroprotection in older adults and aged animal models. A 6‐month aerobic exercise intervention was found to improve cerebrovascular function by increasing cerebral blood flow and reducing vascular resistance, thereby enhancing cognitive performance.^24^ It is reported that exercise‐induced higher CRF is associated with increased functional magnetic resonance imaging (fMRI) activity during associative encoding tasks in key brain regions, including the prefrontal cortex, thalamus, and hippocampus.^25^ Notably, older adults with high CRF, who maintain higher exercise capacity, showed fMRI activation more similar to young adults in associative encoding regions.^25^ Additionally, 20 weeks of treadmill exercise improved spatial memory in aged rats, an effect associated with increased BDNF levels and reduced activation of microglia.^26^ In terms of neuroprotective mechanisms, even a brief 10‐day treadmill exercise regimen normalized the glycolytic profile of microglia in aged mice, thereby reducing senescence markers.^27^ Furthermore, 8 weeks of treadmill exercise suppressed microglia activation and neuroinflammation by downregulating receptor‐interacting protein kinase 1 (RIPK1)‐mediated NF‐κB and JNK pathways.^6^ Four weeks of aerobic treadmill exercise positively modulated synaptic ultrastructure in the hippocampus.^28^

### 3.5 Effects of exercise on brain structure and function

#### 3.5.1 Neuroplasticity

Exercise significantly promotes neuroplasticity by enhancing synaptic density and neurogenesis. Treadmill exercise has been shown to increase synaptic density in the hippocampus of aged mice. Similarly, aerobic exercise over 10 days increased both synaptic density and neurogenesis in the hippocampus of middle‐aged rats, which was accompanied by a reduction in ERK and p38 activation.^29^ Exercise can significantly reduce neuronal apoptosis, with reduced NF‐κB and miR‐503 expression, and increased BDNF levels in vivo and in oxygen‐glucose deprivation‐treated hippocampal neurons. Mechanistically, NF‐κB/miR‐503 signaling negatively regulated BDNF by directly targeting its expression, and modulation of miR‐503 reversed cognitive and molecular alterations.^30^

#### 3.5.2 Cerebrovascular function

Exercise plays a crucial role in improving cerebrovascular function and promoting angiogenesis. Treadmill exercise promoted angiogenesis and improved neurological function following cerebral ischemia by increasing the expression of membrane‐type 1 matrix metalloproteinase (MT1‐MMP) and reducing reversion‐inducing cysteine‐rich protein with kazal motifs (RECK).^31^ In human studies, 6 months of aerobic exercise improved cerebrovascular function in older adults with increased blood flow velocity in the middle cerebral artery and cerebrovascular conductance index.^24^ Additionally, higher CRF is associated with increased levels of VEGF, which is linked to better cognitive performance, while higher midlife CRF may play a role in preserving middle and medial temporal volumes in late adulthood.^3^ Bello et al. further investigated how a 12‐week aerobic exercise intervention influences cerebrovascular and cognitive function in sedentary older adults aged 65–80 years using a multimodal imaging approach. Participants were randomly assigned to aerobic interval cycling or balance/stretching training, and pre–post assessments included VO2 fitness testing, arterial spin labeling for cerebral perfusion, hypercapnic fMRI for cerebrovascular reactivity (CVR), and task‐based fMRI during verbal fluency and motor tasks. The results suggest that improved cardiovascular fitness is associated with enhanced cortical activation patterns and better cognitive‐motor performance, potentially mediated by increased cerebral perfusion and CVR in frontal and motor‐related brain regions.^32^

#### 3.5.3 Neurotransmitter and neurotrophic factor regulation

Exercise modulates the regulation of key neurotransmitters and neurotrophic factors. It increases the levels of BDNF, thereby supporting neurogenesis and synaptic plasticity.^9^ Exercise increases dopamine levels in the striatum, which contributes to improved motor function.^33^ It also elevates serotonin levels in the hippocampus, leading to improved mood and cognitive function while simultaneously reducing apoptotic signaling, such as p75.^9^

### 3.6 Effects of exercise on mental health

#### 3.6.1 Emotional regulation

Different modalities of exercise exert distinct effects on emotional regulation and mental health. Aerobic exercise has been shown to reduce anxiety‐like behaviors via the mitogen‐activated protein kinase signaling pathway, whereas resistance exercise alleviated depressive‐like behaviors through the PI3K/AKT and neurotrophin pathways.^34^ Additionally, 8 weeks of treadmill exercise reduced depressive‐like behaviors by upregulating BDNF and calmodulin‐dependent protein kinase II (CaMKII).^14^ It is reported that exercise can also alleviate high‐fat diet (HFD)‐induced depressive‐like behaviors in young C57BL/6J mice subjected to 8 weeks of HFD with treadmill exercise. Exercise significantly improved depressive‐like behaviors, restored hippocampal gene expression, and enhanced synaptic plasticity through activation of neuronal autophagy in the hippocampal CA1 region via the Wnt5a/CaMKII signaling pathway.^35^ Overall, exercise regulates serotonin and dopamine levels and promotes neuroplasticity, contributing to enhanced emotional well‐being.^2^, ^9^

#### 3.6.2 Cognitive reserve and psychological resilience

Regular exercise significantly enhances cognitive reserve and psychological resilience by modulating inflammatory responses and improving adaptability. A 6‐week aerobic exercise program reduced pro‐inflammatory markers, such as IL‐6, and increased anti‐inflammatory markers, such as IL‐10, which collectively improved mood and reduced stress.^36^ Twelve weeks of treadmill exercise combined with virtual reality improved cognitive flexibility and reduced both stress and fall rates in older adults.^17^ Ultimately, regular exercise provides long‐term mental health benefits by promoting neuroplasticity and mitigating inflammation.^27^, ^36^

### 3.7 Exercise as an intervention for neurodegenerative diseases

#### 3.7.1 AD

Exercise serves as a potent intervention for AD by targeting amyloid pathology and neuroinflammation. Treadmill exercise significantly reduced soluble amyloid‐beta levels and enhanced synaptic plasticity in aged APP/PS1 mice.^37^ Long‐term treadmill exercise also improved spatial memory, increased the number of BDNF‐positive cells, and reduced the presence of activated microglia in AD mouse models.^26^ Furthermore, exercise mitigates neuroinflammation by downregulating RIPK1‐mediated NF‐κB and JNK pathways^6^ and normalizes the glycolytic profile of microglia, thereby offering neuroprotective benefits.^27^

#### 3.7.2 PD

Exercise interventions have demonstrated significant efficacy in alleviating symptoms of PD. A 12‐week program of treadmill exercise combined with virtual reality improved dual‐task gait performance and cognitive flexibility in PD patients.^17^ In animal models, treadmill exercise increased dopamine levels, which improved spontaneous locomotion and motor function in a progressive 1‐methyl‐4‐phenyl‐1,2,3,6‐tetrahydropyridine (MPTP)‐induced model of PD. Additionally, exercise reduced markers of oxidative stress and inflammation, further supporting its therapeutic potential for PD.^33^

### 3.8 Molecular mechanisms

#### 3.8.1 Single‐molecule mechanisms

The benefits of exercise are mediated by various single‐molecule mechanisms involving neurotrophic factors, neurotransmitters, and inflammatory markers. Exercise upregulates BDNF, which supports neurogenesis and synaptic plasticity. For example, 26 weeks of treadmill exercise increased plasma Cathepsin B (CTSB) and BDNF levels, which correlated with improved cognition.^38^ Running also increases the expression of BDNF and doublecortin (DCX) in hippocampal cells.^39^ Additionally, exercise promotes the expression of VEGF, which is crucial for angiogenesis and cerebral blood flow.^40^ Higher CRF attenuated increases in WMH,^3^ and treadmill exercise promoted angiogenesis post‐ischemia via the upregulation of MT1‐MMP.^31^ Exercise modulates the dopaminergic and serotonergic systems, with distinct exercise types regulating mood disorders through different hippocampal gene expression patterns.^34^ Exercise also suppresses neuroinflammation via RIPK1‐mediated pathways.^6^ Finally, exercise reduces pro‐inflammatory cytokines such as IL‐6 and TNF‐α. For instance, 4 weeks of aerobic exercise improved memory and reduced neuroinflammation via the BDNF/FNDC5/irisin and Nrf2/HO‐1 pathways,^41^ while 8 weeks of treadmill exercise improved memory and gut microbiota composition by reducing inflammatory cytokines.^8^

#### 3.8.2 Omics approaches

Omics approaches have elucidated the comprehensive molecular changes induced by exercise. Transcriptomic analyses reveal that exercise upregulates genes associated with synaptic plasticity and downregulates those involved in neuroinflammation. For example, 8 weeks of treadmill exercise reversed key gene expression alterations and inhibited the NOD‐like receptor signaling pathway in the hippocampus and prefrontal cortex.^42^ Proteomic studies demonstrate that exercise increases the levels of neuroprotective proteins, such as BDNF and synaptophysin, and 4 weeks of aerobic treadmill exercise positively modulated synaptic ultrastructure.^28^ Metabolomic profiling indicates that exercise alters the brain's metabolic profile by increasing energy metabolites and reducing oxidative stress metabolites, while also upregulating myokines such as IL‐6, IGF‐1, BDNF, CTSB, and irisin.^7^ Lastly, epigenomic research shows that exercise induces epigenetic modifications, including DNA methylation and histone acetylation. For instance, daily moderate exercise increased histone H4 acetylation in aged rats, further highlighting the profound molecular impact of physical activity.^43^

## 4 DISCUSSION

This systematic review synthesizes the extensive evidence demonstrating the profound positive impacts of exercise on cognitive function and brain health across the lifespan. By adhering to PRISMA 2020 guidelines, we provide a robust overview of how various exercise modalities exert neuroprotective effects through structural, functional, and molecular adaptations. Rather than viewing these benefits in isolation, our findings reveal a continuum of exercise‐induced neuroplasticity that operates through universal mechanisms while yielding age‐specific cognitive outcomes.

Neurotrophic factors and omics as universal mechanisms: a cross‐cutting theme across all age groups and exercise modalities is the central role of neurotrophic factors, particularly BDNF, in mediating cognitive benefits. In young adults, acute aerobic exercise upregulates BDNF, facilitating the efficient reallocation of neural resources and improving cognitive flexibility.^2^, ^20^, ^21^ In older adults and animal models of neurodegeneration, this same BDNF upregulation is crucial for preserving spatial memory, enhancing synaptic density, and counteracting neuroinflammation.^26^, ^39^, ^44^ This aligns with foundational neuroscience reviews positing that exercise acts as a fundamental behavioral intervention to enhance brain health via growth factor cascades.^45^ Furthermore, our integration of recent omics data, including transcriptomics, proteomics, and metabolomics, demonstrates that exercise induces lasting cellular changes, such as the upregulation of synaptic plasticity genes and the suppression of NOD‐like receptor signaling pathways.^28^, ^42^ These multi‐omics findings confirm that exercise triggers a systemic, multi‐target molecular response rather than acting through isolated pathways.

From cognitive enhancement to cognitive preservation: when viewed across the lifespan, the impact of exercise transitions from cognitive enhancement to cognitive preservation. In youth, exercise optimizes executive function and multitasking abilities, directly translating to improved academic and occupational performance.^20^ During middle age, the focus shifts to building cognitive reserve; higher cardiorespiratory fitness is strongly associated with preserved gray matter volume in vulnerable regions such as the middle temporal gyrus, effectively delaying the onset of cognitive decline.^3^, ^5^, ^22^ In older adulthood and in the context of neurodegenerative diseases like Alzheimer's and Parkinson's, exercise serves as a potent therapeutic tool to mitigate neuroinflammation, normalize microglial profiles, and maintain spatial cognition.^6^, ^17^, ^27^ This lifespan continuum underscores that regular physical activity is not merely a rehabilitative tool for the elderly but a lifelong requirement for optimal neurological function.

Exercise modality and dose‐response considerations: Although establishing definitive, prescriptive dose‐response relationships remains challenging due to heterogeneity in protocols, the evidence synthesized in this review permits a descriptive mapping of intensity‐duration signals to specific cognitive and molecular outcomes. At the acute end of the spectrum, even brief bouts of moderate aerobic exercise (e.g., 10 min) enhance executive function across age groups and upregulate hippocampal BDNF.^2^, ^4^, ^21^ In the short‐to‐medium term (4–8 weeks), moderate‐intensity aerobic exercise consistently targets neuroinflammatory pathways: 4 weeks attenuates LPS‐induced cognitive dysfunction by reducing oxidative stress and glial activation,^41^ while 8 weeks suppresses microglia activation via RIPK1‐mediated NF‐κB and JNK signaling^6^ and favorably modulates synaptic ultrastructure^28^ and gut microbiota–brain inflammatory axes.^8^ Conversely, HIIT appears to engage distinct molecular pathways that preferentially benefit cognitive flexibility and emotional regulation; accumulated HIIT in aged rodents improved hippocampal memory‐related proteins while reducing anxiety‐ and depressive‐like behaviors,^14^ suggesting that intensity, not merely volume, determines the mechanistic profile. Over longer durations (≥6 months), aerobic exercise drives structural cerebrovascular remodeling, including increased cerebral blood flow and angiogenesis via VEGF and MT1‐MMP upregulation,^3^, ^24^, ^31^ and sustains systemic biomarker changes (e.g., elevated plasma cathepsin B) associated with preserved cognition.^38^, ^44^ Resistance training complements this profile by engaging neuromuscular pathways and upregulating myokines such as IL‐6, IGF‐1, and BDNF that support neuroplasticity.^7^, ^16^ Importantly, combined multimodal programs that integrate aerobic, resistance, and cognitive components appear to harness the benefits of each domain. A recent meta‐analysis of combined aerobic and resistance exercise in middle‐aged and older adults with T2DM suggests that combining aerobic and resistance training yields synergistic cognitive effects beyond single‐modality interventions.^46^ Taken together, these findings suggest that moderate‐intensity exercise may be optimized for anti‐neuroinflammatory goals, whereas HIIT may be preferentially indicated for cognitive flexibility and mood regulation, and long‐term adherence is required for cerebrovascular and structural brain adaptations.

While the mechanistic evidence is compelling, several limitations must be acknowledged. First, the included studies exhibit considerable clinical and methodological heterogeneity. Interventions range from acute 10‐min bouts to 6‐month programs, encompassing various modalities at differing intensities. This heterogeneity precludes the establishment of definitive dose–response relationships and limits the generalizability of specific exercise prescriptions. Second, our literature search was confined to three databases (PubMed, Web of Science, and Scopus); excluding specialized databases such as PsycINFO, CINAHL, and Cochrane CENTRAL may have resulted in the omission of relevant psychological or clinical trial literature. Third, 15 of the 37 included studies (40.5%) focused on molecular mechanisms, predominantly in animal models. While these models provide invaluable molecular insights, they may not perfectly translate to human physiology, presenting a significant translational challenge. Finally, there is a potential for publication bias, as studies reporting positive findings are more likely to be published. Moreover, because our search was limited to three major bibliographic databases and did not encompass gray literature, trial registries, or unpublished dissertations, negative or null results from completed but unreported trials may be underrepresented. This could inflate the apparent magnitude of exercise benefits and should be considered when interpreting the generalizability of our conclusions. Clinically, these findings strongly support the prescription of regular, multimodal physical activity as a preventive and therapeutic tool for cognitive health across all ages. Future research should leverage single‐cell omics to delineate cell‐type‐specific responses to exercise in human cohorts. Furthermore, well‐designed, long‐term longitudinal studies are needed to determine the optimal “dose” of exercise required to maximize cognitive benefits and to understand the long‐term trajectories of exercise‐induced neuroplasticity fully.

## 5 CONCLUSION

Regular physical exercise is a potent, multifaceted intervention that enhances cognitive function and supports brain health from youth through older adulthood. By promoting neuroplasticity, improving cerebrovascular function, and regulating critical molecular pathways, exercise offers a promising defense against age‐related cognitive decline and neurodegenerative diseases. It should be noted, however, that many of the precise molecular mechanisms identified in this review are derived primarily from animal models, and further well‐designed translational studies in humans are needed to confirm these pathways and establish optimal exercise prescriptions. Integrating consistent physical activity into daily life remains a foundational strategy for lifelong neurological well‐being.

## AUTHOR CONTRIBUTIONS

Issam AbuQeis, Abeer Teeti, and Amro Titi contributed to the conceptualization, literature search, data synthesis, and drafting of the manuscript. All authors have read and agreed to the published version of the manuscript.

## CONFLICT OF INTEREST STATEMENT

The authors declare no conflicts of interest.

## ETHICS STATEMENT

Not applicable. This is a literature review and did not involve new human or animal subjects.

## Supporting information

Supporting File 1.

Supporting File 2.

Supporting File 3.

Supporting File 4.

## Data Availability

The data that support the findings of this study are available from the corresponding author upon reasonable request. All data presented in this study are derived from peer‐reviewed literature available via PubMed, Web of Science, or Scopus.
