# Supplementary material for: The impact of exercise on cognitive function and brain health across the lifespan: A systematic review
Source: Ibrain. 2026 Jun 14;12(2):190–200. doi: 10.1002/ibra.70024 (PMC13310238; doi:10.1002/ibra.70024)
Supplement: Supplementary file 3 — Supporting File 3. [file IBRA-12-190-s002.docx]

Complete Search Syntax

**PubMed Search Strategy**

("Exercise"[Mesh] OR "Exercises"[tiab] OR "Exercise, Physical"[tiab] OR "Exercises, Physical"[tiab] OR "Physical Exercise"[tiab] OR "Physical Exercises"[tiab] OR "Exercise, Isometric"[tiab] OR "Exercises, Isometric"[tiab] OR "Isometric Exercises"[tiab] OR "Isometric Exercise"[tiab] OR "Exercise, Aerobic"[tiab] OR "Aerobic Exercise"[tiab] OR "Aerobic Exercises"[tiab] OR "Exercises, Aerobic"[tiab] OR "Exercise Training"[tiab] OR "Exercise Trainings"[tiab] OR "Training, Exercise"[tiab] OR "Trainings, Exercise"[tiab] OR "Physical Activity"[tiab] OR "Activities, Physical"[tiab] OR "Activity, Physical"[tiab] OR "Physical Activities"[tiab] OR "Active Breaks"[tiab] OR "Activity Breaks"[tiab] OR "Acute Exercise"[tiab] OR "Acute Exercises"[tiab] OR "Exercise, Acute"[tiab] OR "Exercises, Acute"[tiab])
AND
("Cognition"[Mesh] OR "Cognitive Dysfunction"[Mesh] OR "Cognitions"[tiab] OR "Cognitive Function"[tiab] OR "Cognitive Functions"[tiab] OR "Function, Cognitive"[tiab] OR "Functions, Cognitive"[tiab] OR "Insight"[tiab] OR "Insights"[tiab] OR "Cognitive Dysfunctions"[tiab] OR "Dysfunction, Cognitive"[tiab] OR "Dysfunctions, Cognitive"[tiab] OR "Cognitive Disorder"[tiab] OR "Cognitive Disorders"[tiab] OR "Disorder, Cognitive"[tiab] OR "Disorders, Cognitive"[tiab] OR "Cognitive Impairments"[tiab] OR "Cognitive Impairment"[tiab] OR "Impairment, Cognitive"[tiab] OR "Impairments, Cognitive"[tiab] OR "Mild Cognitive Impairment"[tiab] OR "Cognitive Impairment, Mild"[tiab] OR "Cognitive Impairments, Mild"[tiab] OR "Impairment, Mild Cognitive"[tiab] OR "Impairments, Mild Cognitive"[tiab] OR "Mild Cognitive Impairments"[tiab] OR "Cognitive Decline"[tiab] OR "Cognitive Declines"[tiab] OR "Decline, Cognitive"[tiab] OR "Declines, Cognitive"[tiab] OR "Mental Deterioration"[tiab] OR "Deterioration, Mental"[tiab] OR "Deteriorations, Mental"[tiab] OR "Mental Deteriorations"[tiab] OR "brain health"[tiab])
AND
("Age Groups"[Mesh] OR "Age Group"[tiab] OR "Group, Age"[tiab] OR "lifespan"[tiab] OR "life span"[tiab])

**Scopus Search Strategy**

(TITLE-ABS-KEY("Exercise") OR TITLE-ABS-KEY("Exercises") OR TITLE-ABS-KEY("Exercise, Physical") OR TITLE-ABS-KEY("Exercises, Physical") OR TITLE-ABS-KEY("Physical Exercise") OR TITLE-ABS-KEY("Physical Exercises") OR TITLE-ABS-KEY("Exercise, Isometric") OR TITLE-ABS-KEY("Exercises, Isometric") OR TITLE-ABS-KEY("Isometric Exercises") OR TITLE-ABS-KEY("Isometric Exercise") OR TITLE-ABS-KEY("Exercise, Aerobic") OR TITLE-ABS-KEY("Aerobic Exercise") OR TITLE-ABS-KEY("Aerobic Exercises") OR TITLE-ABS-KEY("Exercises, Aerobic") OR TITLE-ABS-KEY("Exercise Training") OR TITLE-ABS-KEY("Exercise Trainings") OR TITLE-ABS-KEY("Training, Exercise") OR TITLE-ABS-KEY("Trainings, Exercise") OR TITLE-ABS-KEY("Physical Activity") OR TITLE-ABS-KEY("Activities, Physical") OR TITLE-ABS-KEY("Activity, Physical") OR TITLE-ABS-KEY("Physical Activities") OR TITLE-ABS-KEY("Active Breaks") OR TITLE-ABS-KEY("Activity Breaks") OR TITLE-ABS-KEY("Acute Exercise") OR TITLE-ABS-KEY("Acute Exercises") OR TITLE-ABS-KEY("Exercise, Acute") OR TITLE-ABS-KEY("Exercises, Acute"))
AND
(TITLE-ABS-KEY("Cognition") OR TITLE-ABS-KEY("Cognitive Dysfunction") OR TITLE-ABS-KEY("Cognitions") OR TITLE-ABS-KEY("Cognitive Function") OR TITLE-ABS-KEY("Cognitive Functions") OR TITLE-ABS-KEY("Function, Cognitive") OR TITLE-ABS-KEY("Functions, Cognitive") OR TITLE-ABS-KEY("Insight") OR TITLE-ABS-KEY("Insights") OR TITLE-ABS-KEY("Cognitive Dysfunctions") OR TITLE-ABS-KEY("Dysfunction, Cognitive") OR TITLE-ABS-KEY("Dysfunctions, Cognitive") OR TITLE-ABS-KEY("Cognitive Disorder") OR TITLE-ABS-KEY("Cognitive Disorders") OR TITLE-ABS-KEY("Disorder, Cognitive") OR TITLE-ABS-KEY("Disorders, Cognitive") OR TITLE-ABS-KEY("Cognitive Impairments") OR TITLE-ABS-KEY("Cognitive Impairment") OR TITLE-ABS-KEY("Impairment, Cognitive") OR TITLE-ABS-KEY("Impairments, Cognitive") OR TITLE-ABS-KEY("Mild Cognitive Impairment") OR TITLE-ABS-KEY("Cognitive Impairment, Mild") OR TITLE-ABS-KEY("Cognitive Impairments, Mild") OR TITLE-ABS-KEY("Impairment, Mild Cognitive") OR TITLE-ABS-KEY("Impairments, Mild Cognitive") OR TITLE-ABS-KEY("Mild Cognitive Impairments") OR TITLE-ABS-KEY("Cognitive Decline") OR TITLE-ABS-KEY("Cognitive Declines") OR TITLE-ABS-KEY("Decline, Cognitive") OR TITLE-ABS-KEY("Declines, Cognitive") OR TITLE-ABS-KEY("Mental Deterioration") OR TITLE-ABS-KEY("Deterioration, Mental") OR TITLE-ABS-KEY("Deteriorations, Mental") OR TITLE-ABS-KEY("Mental Deteriorations") OR TITLE-ABS-KEY("brain health"))
AND
(TITLE-ABS-KEY("Age Groups") OR TITLE-ABS-KEY("Age Group") OR TITLE-ABS-KEY("Group, Age") OR TITLE-ABS-KEY("lifespan") OR TITLE-ABS-KEY("life span"))

**Web of Science Search Strategy**

TS=("Exercise" OR "Exercises" OR "Exercise, Physical" OR "Exercises, Physical" OR "Physical Exercise" OR "Physical Exercises" OR "Exercise, Isometric" OR "Exercises, Isometric" OR "Isometric Exercises" OR "Isometric Exercise" OR "Exercise, Aerobic" OR "Aerobic Exercise" OR "Aerobic Exercises" OR "Exercises, Aerobic" OR "Exercise Training" OR "Exercise Trainings" OR "Training, Exercise" OR "Trainings, Exercise" OR "Physical Activity" OR "Activities, Physical" OR "Activity, Physical" OR "Physical Activities" OR "Active Breaks" OR "Activity Breaks" OR "Acute Exercise" OR "Acute Exercises" OR "Exercise, Acute" OR "Exercises, Acute")
AND
TS=("Cognition" OR "Cognitive Dysfunction" OR "Cognitions" OR "Cognitive Function" OR "Cognitive Functions" OR "Function, Cognitive" OR "Functions, Cognitive" OR "Insight" OR "Insights" OR "Cognitive Dysfunctions" OR "Dysfunction, Cognitive" OR "Dysfunctions, Cognitive" OR "Cognitive Disorder" OR "Cognitive Disorders" OR "Disorder, Cognitive" OR "Disorders, Cognitive" OR "Cognitive Impairments" OR "Cognitive Impairment" OR "Impairment, Cognitive" OR "Impairments, Cognitive" OR "Mild Cognitive Impairment" OR "Cognitive Impairment, Mild" OR "Cognitive Impairments, Mild" OR "Impairment, Mild Cognitive" OR "Impairments, Mild Cognitive" OR "Mild Cognitive Impairments" OR "Cognitive Decline" OR "Cognitive Declines" OR "Decline, Cognitive" OR "Declines, Cognitive" OR "Mental Deterioration" OR "Deterioration, Mental" OR "Deteriorations, Mental" OR "Mental Deteriorations" OR "brain health")
AND
TS=("Age Groups" OR "Age Group" OR "Group, Age" OR "lifespan" OR "life span")
